# High prevalence of *Toxoplasma gondii* infection in free-roaming dogs from Ecuador: a proxy for sentinel surveillance of zoonotic threats in low- and middle-income countries

**DOI:** 10.3389/fvets.2025.1672769

**Published:** 2025-10-02

**Authors:** Darwin Paredes-Núñez, Andrés Esteban Barragán-Peña, Ángel Sebastián Rodríguez-Pazmiño, Alberto Velez, Marylin Cruz, Mauricio Xavier Salas-Rueda, Alexandra Narváez, Solón Alberto Orlando, Miguel Angel Garcia-Bereguiain

**Affiliations:** ^1^One Health Research Group, Universidad de Las Américas, Quito, Ecuador; ^2^Agencia de Regulación y Control para la Bioseguridad de Galápagos, Puerto Ayora, Ecuador; ^3^Universidad Politécnica Salesiana, Cuenca, Ecuador; ^4^Universidad Espíritu Santo, Guayaquil, Ecuador; ^5^Instituto Nacional de Salud Pública e Investigación, Guayaquil, Ecuador; ^6^Universidad Ecotec, Guayaquil, Ecuador

**Keywords:** toxoplasma gondii, seropositivity, toxoplasmosis, ELISA, dogs, zoonotic diseases, sentinel surveillance, one health

## Abstract

Toxoplasmosis is a globally distributed zoonotic disease caused by the protozoan parasite *Toxoplasma gondii*. Although dogs are not definitive hosts, they can act as environmental sentinels for the risk of toxoplasmosis in humans. In this study, we estimated the seropositivity of *T. gondii* in stray dogs from Ecuador for the first time and assessed differences in prevalence across regions and between urban and rural settings. A total of 272 free-roaming dogs from the four main regions of Ecuador (Andean, Coastal, Amazon, and Insular regions) were included in this study. Serum samples were collected and tested using a commercial indirect ELISA kit for the detection of *T. gondii* antibodies. An overall high seropositivity of 39.7% (95% CI: 33.9–45.5%) was observed, with no significant differences between regions or urban and rural zones. This is the first large-scale serological survey of *T. gondii* in Ecuadorian dogs, confirming widespread environmental exposure to the parasite in the country. Given the large population of free-roaming dogs and the high seropositivity of *T. gondii*, integrated One Health strategies are needed, including improved stray animal management, public education on responsible pet ownership, and environmental control measures to mitigate the risk of toxoplasmosis and related diseases in Ecuador.

## 1 Introduction

*Toxoplasma gondii* is a globally distributed, obligate intracellular zoonotic protozoan and the etiological agent of toxoplasmosis. It can infect all warm-blooded vertebrates, including birds (1). *T. gondii* has a life cycle including a sexual cycle within a feline definitive host and an asexual cycle with a wide range of intermediate avian and mammal hosts. Sporulated oocysts are produced within the intestines of felines and shed with their feces into the environment (2). These oocysts are highly resistant and can remain viable in the environment for months, representing a significant source of infection. Transmission occurs through the ingestion of contaminated water or food, or by consuming infected animal tissue (3, 4). When ingested by intermediate hosts, oocysts develop into infective tachyzoites that penetrate host tissue to form cysts of slow-growing bradyzoites. Infection of the definitive or intermediate host may also happen through the ingestion of tissue cysts of infected animals (2–4).

Toxoplasmosis is a zoonotic disease with a high impact on human and animal health. Although most cases are asymptomatic, toxoplasmosis may cause severe outcomes, including stillbirth in humans and animals. In this sense, it is a serious public health issue impacting human reproduction, but it also affects animal production and wildlife conservation. In dogs, toxoplasmosis is rarely a primary disease and is generally associated with immunosuppression, co-infection with other pathogens, and the absence of vaccination against canine distemper virus (CDV) (5). Dogs act as intermediate hosts, acquiring the infection primarily through the ingestion of raw meat contaminated with tissue cysts (bradyzoites). After infection, most dogs remain asymptomatic and do not develop pathological lesions; however, in severe cases, neurological, pulmonary, muscular, and digestive impairments may be observed (4). Although dogs do not excrete oocysts in their feces, they play a role in the epidemiology of toxoplasmosis as mechanical vectors, transporting them on their fur or paws, which represents a potential zoonotic risk due to their close interaction with humans (5–7).

The diagnosis of *T. gondii* is primarily based on serological tests, including indirect immunofluorescence (IFAT), agglutination test (AT), and enzyme-linked immunosorbent assay (ELISA) (3, 8). Among these, the ELISA technique is widely used due to its high sensitivity and specificity. In particular, the indirect ELISA is the method of choice for detecting this pathogen, as it allows for the identification of both IgG and IgM antibodies in the host, providing key information on exposure and the animal’s immunological status (3, 9).

As mentioned above, the evaluation of toxoplasmosis seroprevalence in dogs has remarkable epidemiological and clinical importance as a proxy for estimating the risk of human infection. Several studies have shown that toxoplasmosis seropositivity in dog populations is related to exposure levels in humans (5, 7, 10). It is estimated that approximately one-third of the global population carries the parasite in a latent form, and the burden of toxoplasmosis is higher in low and middle-income countries (LMICs) due to factors such as poor water quality and inadequate hygiene (11–14). In this sense, large populations of free-roaming dogs are also more frequent in LMICs like Ecuador and have been associated with risk of zoonotic transmission (15–21), highlighting the need for a comprehensive approach from a One Health perspective to manage diseases like rabies (15), leptospirosis (18–20), brucellosis, or Q-fever (17).

This study aimed to determine the seroprevalence of *T. gondii* in free-ranging canines from various regions of Ecuador and to identify geographical risk factors. Given the lack of prior epidemiological data in this canine population, the present study provides an essential baseline for future research.

## 2 Methodology

### 2.1 Study design

**Figure 1:**
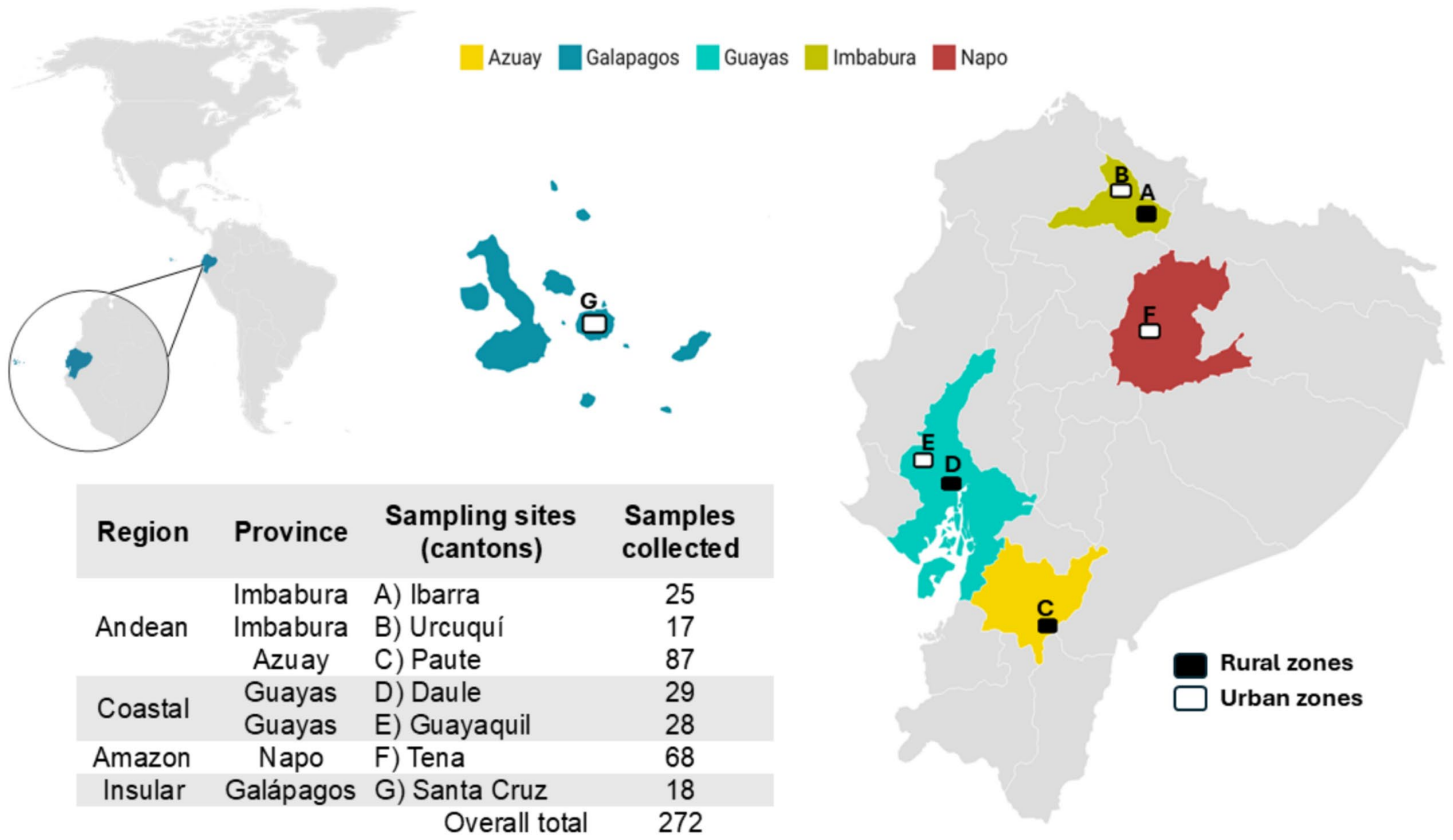
Distribution of free-roaming dogs included in the study by region and canton in Ecuador. Black and white boxes represent urban and rural zones, respectively.

This cross-sectional study was conducted using a convenience sample of stray dogs selected during spay and neuter campaigns carried out in 2018 and 2019. A total of 272 dogs were included in the study. The dogs were distributed across the following provinces and cantons (Figure 1): 42 dogs from Imbabura province (Andean region; 25 from Ibarra canton and 17 from Urcuquí canton); 87 dogs from Azuay province (Andean region; 87 dogs from Paute canton); 57 dogs from Guayas province (Coastal region; 29 from Daule canton and 28 from Guayaquil canton); 68 dogs from Napo province (Amazon region; Tena canton); and 18 dogs from Galapagos province (Insular region; Santa Cruz canton).

### 2.2 Sample collection

With the assistance of a veterinarian, 5 mL of blood was collected from the cephalic vein of each dog using red-top tubes containing clot activator for serum separation. The samples were then stored in a refrigerated container and transported to the laboratory for analysis. After clotting, 1 mL of serum was extracted and transferred to a 1.5 mL Eppendorf tube. Finally, the serum samples were stored at −20 °C until further processing and analysis.

### 2.3 Serology

The commercial indirect ELISA kit “ID Screen® Toxoplasmosis Indirect Multi-species” (IDVet, France) was used for the detection of antibodies against the P30 protein of *T. gondii* in canine serum samples. The assay was performed according to the manufacturer’s instructions, analyzing all 272 samples along with positive and negative controls included in each test run.

### 2.4 Statistical analysis

A statistical analysis was performed using the chi-squared test (χ^2^) and Fisher’s exact test to evaluate potential differences in the frequency of *T. gondii* infection in dogs, considering their location (urban vs. rural areas), geographic region, and canton. For these analyses, contingency tables were constructed using the numbers of seropositive and seronegative animals within each category. Statistical significance was defined at a *p*-value < 0.05. Additionally, 95% confidence intervals (95% CI) were calculated for overall seroprevalence, as well as for seroprevalence within each geographic region and canton. Due to limited sample sizes in certain regions (e.g., the Galapagos region, where only one canton was sampled), specific confidence interval estimations may present limitations related to small sample sizes. All statistical analyses were performed using R software (R version 4.4.3 (2025-02-28)).

## 3 Results

For this study, 272 serum samples were collected from free-roaming dogs originating from both urban and rural areas of Ecuador, covering the country’s four geographical regions (Andean, Coastal, Amazon, and Insular). The samples were analyzed using an ELISA assay designed to detect antibodies against the P30 protein of *T. gondii*. Veterinarians reported no clinical signs for any of the dogs included in the study.

Of the 272 stray dogs tested, 108 were positive for antibodies against *T. gondii*, corresponding to an overall seroprevalence of 39.7% (95% CI: 33.9–45.5%). Table 1 shows the distribution of seropositivity values according to geographic region, canton and urban/rural location.

**Table 1:** Seroprevalence of *Toxoplasma gondii* in free-roaming dogs from Ecuador, distributed according to geographic region, canton, and urban or rural zones.

Region	Canton	*T. gondii* seropositivity		
		% Urban (n/total)	% Rural (n/total)	Total % (n/total; 95% CI)
Andean	Ibarra	40% (10/25) (95% CI: 21.8–61.1%)	-	35.7% (46/129) (95% CI: 27.6–44.6%)
	Paute	-	29.9% (26/87) (95% CI: 20.8–40.8%)	
	Urcuquí	-	58.8% (10/17) (95% CI: 33.5–80.6%)	
Coastal	Daule	-	44.8% (13/29) (95% CI: 26.95–64.02%)	42.1% (24/57) (95% CI: 29.4–55.9%)
	Guayaquil	39.3% (11/28) (95% CI: 22.1–59.3%)	-	
Amazon	Tena	-	41.2% (28/68) (95% CI: 29.6–53.8%)	41.2% (28/68) (95% CI: 29.6–53.8%)
Insular	Santa Cruz	-	55.6% (10/18) (95% CI: 31.4–77.6%)	55.6% (10/18) (95% CI: 31.4–77.6%)
Total % (n/total) (95% CI)		39.6% (21/53) (95% CI: 26.8–53.9%)	39.7% (87/219) (95% CI: 33.3–46.6%)	39.7% (108/272) (95% CI: 33.9–45.8%)

The seropositivity values by canton were Ibarra (40%), Urcuquí (58.8%), Paute (29.9%), Daule (44.8%), Guayaquil (39.3%), Tena (41.2%), and Santa Cruz (55.6%). The seropositivity values for the Andean, Coastal, Amazon, and Galapagos regions were 35.7, 42.1, 41.2, and 55.6%, respectively. Regarding urban and rural locations, the seropositivity values were 39.6 and 39.7%, respectively. The chi-square and Fisher’s exact tests yielded a *p*-value of 1 for the comparison between urban and rural areas. The chi-square yielded *p*-values of 0.21 and 0.40 for the comparison between the four regions, and a *p*-value of 0.21 for the comparison between the seven cantons. Overall, no significant differences were found for the toxoplasmosis prevalence values between urban and rural areas, cantons, or regions.

## 4 Discussion

This study reveals a high level of environmental exposure to *T. gondii* among free-ranging canines across various geographical regions of Ecuador, including the Insular region (Galapagos Islands). The overall seroprevalence of 39.7% for free-roaming dogs’ toxoplasmosis suggests that this zoonotic disease would be a significant public health threat in Ecuador. Uncontrolled stray dog populations facilitate the transmission of multiple zoonoses, beyond toxoplasmosis, including rabies, leptospirosis, and intestinal parasites (15, 19, 22). Likewise, the expected interaction of stray dogs with other domestic animals and wildlife (15–21) endorsed a widespread distribution of *T. gondii* and other pathogens across warm-blooded animal species, representing a potential threat to animal production and wildlife conservation (23). Additionally, stray dogs often share habitats with free-ranging domestic and wild felids, the definitive hosts responsible for shedding oocysts into the environment (24). Such contact not only exposes dogs to infection but also reflects broader environmental contamination (25).

Diverse findings have already been reported in other Latin American countries. For instance, seroprevalences ranging from 16 to 61.7% have been reported in either domestic or free-ranging canines from Mexico, Brazil, Panama, and Peru (25–29). Interestingly, those studies found high prevalence of toxoplasmosis not only in free-roaming dogs but also in domestic dogs, endorsing the role of stray dogs as a mechanical vector for the spread of *T. gondii* to domestic animals and humans. Moreover, in our study, no statistically significant differences were observed between the rural and urban settings, underscoring the role of both feral and synanthropic feline reservoirs in the epidemiology of toxoplasmosis in Ecuador, as it has also been reported in China (30).

Our findings highlight the role of stray dogs as sentinel animals in detecting environmental contamination by *T. gondii* oocysts, as previously proposed (31). Other studies have demonstrated a direct correlation between canine toxoplasmosis seropositivity and environmental exposure within human communities, reinforcing their value as indicators of zoonotic risk (31). In this scenario, our results emphasize the importance of a One Health approach to managing toxoplasmosis and other zoonotic diseases in LMICs where large populations of free-roaming dogs serve as zoonotic reservoirs that warrant consideration for sentinel surveillance (17–20).

In this study, we found a high prevalence of toxoplasmosis in free-ranging canines across all locations and geographical regions analyzed, with no statistical significance difference between locations. Beyond its impact on public health and animal production, toxoplasmosis poses a threat to wildlife conservation. In this sense, we call attention to the fact that a very high toxoplasmosis prevalence of over 50% was found in free-roaming dogs from the Santa Cruz Island in the Galapagos. On the other hand, a recent study has reported a high prevalence of antibodies against *T. gondii* in Galapagos sea lion (*Zalophus wollebaeki*), including dead pups (32). *Z. wollebaeki* is an endemic and iconic species of the islands, very present in the main towns, where interactions with stray dogs are common. In this sense, free-roaming dogs could be a mechanical vector for the transmission of toxoplasmosis to sea lions, as well as other diseases, such as canine distemper virus (33). While free-ranging canines pose a problem in Ecuador, affecting public health and animal production, they also pose a threat to wildlife in the iconic Galapagos Islands, where efforts to eradicate them should be implemented.

These risks are further exacerbated by the documented burden of canine vector-borne parasites with spillover potential to native mammals, as well as the widespread environmental exposure of endemic birds to *T*. *gondii* (34, 35). Moreover, free-roaming dogs have been associated with the dissemination of gastrointestinal parasites such as *Ancylostoma caninum* and *Toxocara canis*, which contaminate beaches and recreational areas, thereby exposing human populations to larva migrans and other parasitic zoonoses (22). In light of these combined threats, stricter biosecurity measures and companion animal management strategies, including sterilization, vaccination, registration, and roaming restrictions, are urgently required in inhabited zones and park buffer areas, in accordance with the “Agencia de Regulación y Control de la Bioseguridad y Cuarentena para Galápagos” guidelines (36).

The main limitation of our study was the use of a convenience sample. In this sense, toxoplasmosis prevalence values should be interpreted with caution, as sampling bias cannot be totally ruled out. Nevertheless, the uniform high prevalence values found across the different sample locations support the countrywide widespread occurrence of *T. gondii* in free-roaming dogs from Ecuador. Additionally, it is worth noting that our country faces other challenges in monitoring these diseases, including logistical issues (such as capture, costs, and geography) and inadequate, minimal, or nonexistent support from public institutions. Furthermore, with hundreds of thousands of abandoned dogs living on the streets and a total population of several million (37–39), it is challenging to obtain representative data; however, this does not detract from the importance of this type of study.

In conclusion, free-roaming dogs have a high prevalence of toxoplasmosis in Ecuador, pointing out the threat of transmission to humans or other domestic or wild animals. A comprehensive One Health approach is recommended to control toxoplasmosis in Ecuador, where these animals should be considered as a target population for sentinel surveillance.

## Data Availability

The original contributions presented in the study are included in the article/supplementary material, further inquiries can be directed to the corresponding author.
